# Consensus on the use of artificial intelligence in the management and measurement of vestibular schwannomas: A protocol for a modified delphi consensus

**DOI:** 10.1007/s00234-026-04063-z

**Published:** 2026-06-24

**Authors:** Keng Siang Lee, Steve Connor, Navodini Wijethilake, Tom Vercauteren, Rupert Obholzer, Kazumi Chia, Henricus Kunst, James Tysome, Nick Thomas, Jonathan Shapey

**Affiliations:** 1 Department of Neurosurgery, National Neuroscience Institute, Singapore, Singapore; 2 School of Biomedical Engineering & Imaging Sciences, King’s College London, London, United Kingdom; 3 Department of Neurosurgery, King’s College Hospital, London, United Kingdom; 4 Department of Neuroradiology, King’s College Hospital, London, United Kingdom; 5 Department of Otolaryngology, Guy’s Hospital, London, United Kingdom; 6 Department of Oncology, Guy’s Hospital, London, United Kingdom; 7 Department of Otorhinolaryngology, Dutch Academic Alliance Skull Base Pathology, Radboud University Medical Center, Nijmegen, Netherlands; 8 Department of Otorhinolaryngology, Dutch Academic Alliance Skull Base Pathology, Maastricht University Medical Center+, Maastricht, Netherlands; 9 Department of Otolaryngology, Cambridge University Hospitals, Cambridge, United Kingdom

**Keywords:** Artificial intelligence, Delphi consensus, Vestibular schwannoma, Magnetic resonance, Acquisition, Growth, Measurement, Systematic review

## Abstract

**Introduction:**

The assessment of vestibular schwannomas (VS) requires a standardized approach as growth is a key element in defining treatment strategy. Volumetric measurements offer higher sensitivity and precision, but existing methods of image segmentation, are labour-intensive and prone to variability. Artificial intelligence (AI) frameworks to segment VS using magnetic resonance imaging (MRI) achieving state-of-the-art capability can fully automate the detection and segmentation of VS. These tools can be used for automating the extraction process of various linear and volumetric measurements. A consistent approach to recording data, facilitated by AI, will allow the accumulation and comparison of evidence to identify the most effective treatments for patients with VS.

**Aims:**

This protocol aims to develop a Delphi consensus for the assessment of VS and deployment of AI-based image analysis as a tool for use in VS management.

**Methods:**

A three-phase consensus study will be undertaken; Phase 1: systematic review (PROSPERO registration number CRD42024604452) of trials and observational studies reporting the measurement of VS to identify a list of candidate indicators; Phase 2: refinement of this list and development of a set of questionnaire questions performed by our local steering committee; and Phase 3: a two-round Delphi questionnaire and consensus meeting with expert stakeholders from the British Skull Base Society (BSBS), European Skull Base Society (ESBS) and European Society of Head and Neck Radiology (ESHNR).

**Ethics and dissemination:**

Participants will be recruited through professional bodies. The core reporting set will be disseminated through peer-reviewed publication, co-production with journal editors, research funders and professional bodies, and presentation at national conferences.

**Clinical trial number:**

Not applicable.

## Strengths of the study

- This protocol was co-produced by a multidisciplinary panel with expertise in diagnosing and managing vestibular schwannoma (VS).
- We will work closely with multidisciplinary stakeholders who will be identified through professional bodies such as the British Skull Base Society (BSBS), European Skull Base Society (ESBS) and European Society of Head and Neck Radiology (ESHNR), to participate in the Delphi consensus, and facilitate dissemination.
- A UK and European focus aims to achieve a pragmatic consensus to support research and facilitate early clinical adoption and impact.

## Introduction

Vestibular schwannoma (VS) is a benign intracranial tumour originating from peripheral myelinating Schwann cells within the vestibular division of the vestibulocochlear nerve. The advent availability of magnetic resonance imaging (MRI) has led to an increased prevalence of small asymptomatic VS being detected and patients typically require a period of surveillance [1]. Modern incidence rates suggest that the lifetime risk of developing a sporadic VS likely exceeds one in 500 individuals [2]. The assessment of VS growth is a key element in defining its treatment strategy [3]. The 2003 International Consensus Meeting on Systems for Reporting Results in VS advocates defining size of the VS by its maximal linear diameter on MRI [4]. However, a wide margin of error has to be applied when using linear measurements to guide clinical management. This often results in more frequent surveillance scanning. Linear measurements can also give the impression of intermittent growth, which complicates clinical decision-making. On the contrary, volumetric measurement is a more sensitive and precise method of calculating the true size of the VS and is superior at detecting subtle growth [5, 6]. Nonetheless, current techniques such as manual or semi-automated tumour segmentation on MRI are time-consuming, lack standardisation and are susceptible to inter-observer variability [7]. Furthermore, the limited availability of specialised software tools has posed a barrier to the routine clinical implementation of volumetric assessment [8].

To manage this implementation gap, fully automated artificial intelligence (AI) frameworks to segment VS using MRI have been developed [9], including some that can delineate and differentiate the tumour’s intra- and extra-meatal components [10]. However, an evidence-based, objective measurement standard for VS is required to ensure a consistent approach to inform clinical decision making, facilitate evidence synthesis and support future research in this area. Importantly, the MRI acquisition(s) used to image patients with VS are crucial in determining the accuracy of AI based tools. We outline a modified Delphi consensus involving members of the British Skull Base Society (BSBS), European Skull Base Society (ESBS) and European Society of Head and Neck Radiology (ESHNR), exploring the requirements for VS imaging, assessment and measurement, and the barriers to introducing AI tools to aid in the management of VS.

## Methods

### Study design

**Fig. 1:**
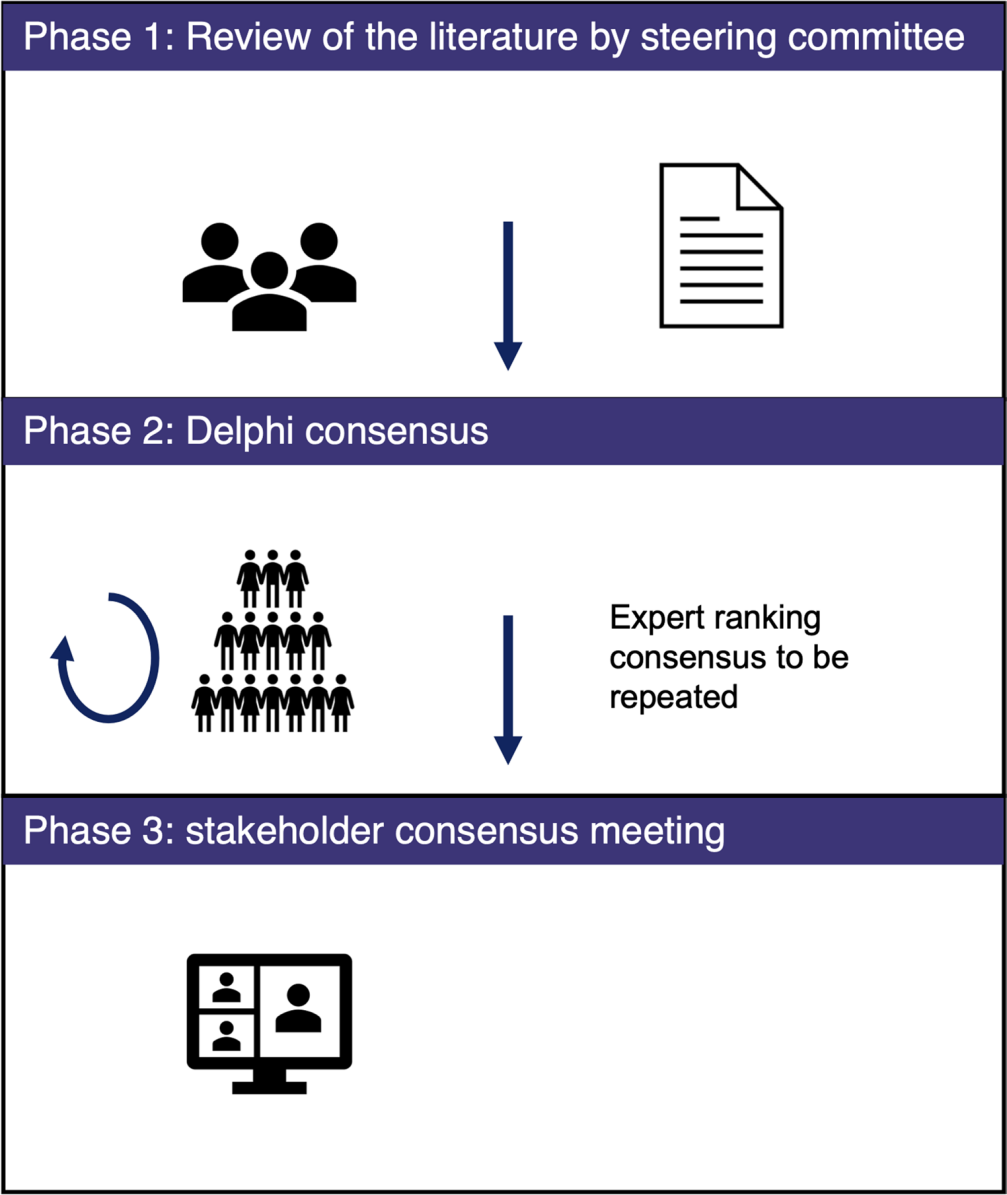
Workflow of the modified Delphi’s process, highlighting the generation of a core measurement set through iterative consensus from expert members of the British Skull Base Society (BSBS), European Skull Base Society (ESBS) and European Society of Head and Neck Radiology (ESHNR)

The study was approved by the King's College London institutional review board (MRA-24/25-51552) and will follow the Accurate Consensus Reporting Document (ACCORD) reporting guideline for consensus methods developed via a modified Delphi. This study was conducted in three phases using methods identified from the development of core outcome sets of randomized trials [11] The workflow of this process is summarised in Fig. 1.

### Phase 1: systematic review for domain generation

A systematic review was undertaken to identify potential indicators used in current clinical practice and research for the (1) MRI acquisition or evaluation of (2) measurement or (3) growth of VS. The systematic review was conducted according to the Preferred Reporting Items for Systematic Reviews and Meta-Analyses (PRISMA) guidelines [12], and was registered on the PROSPERO international prospective register of systematic reviews (registration number CRD42024604452) [ 13–15]. Clinical trial number: not applicable.

Searches of the following three electronic databases were undertaken: Ovid Medline, Ovid Embase, and Cochrane Central Register of Controlled Trials (CENTRAL). Searches were performed in each database from its inception until 4th October 2024. The concepts of “MRI”, “acquisition”, “measurement”, “growth” and “VS”, were used in addition to synonyms and related terms. The full search strategy used for the databases is presented in Table 1. This work highlighted substantial variation in the MRI acquisition used to image VS and the methods used for evaluating measurement and growth of VS, but provided an initial set of indicators for the Delphi process. The core group reviewed the complete list of domain indicators indicated from the systematic review. Through iterative rounds of internal discussion, an initial framework structure was agreed upon including terminology, domains and associated guidance statements. The resulting list was developed into questionnaire items (Tables 2 and 3) [16].

**Table 1:** Search strategy used for the three electronic databases 4 October 2024

No.	Search term
EMBASE search	1998 articles
Vestibular schwannoma concept	
1	exp acoustic neuroma/
2	(vestibular schwannoma* or acoustic neuroma*).tw.
3	1 or 2
MRI concept	
4	exp nuclear magnetic resonance imaging/
5	(magnetic resonance imaging or MRI).tw.
6	4 or 5
Outcome concept	
7	(linear or rate or progression or growth or analysis or volume* or 3D* or size or segment* or diameter* or acquisition or sequence* or measure*).tw.
Combined concepts	
8	3 and 6 and 7
9	limit 8 to yr="2014 -Current"
OVID Medline search	995 articles
Vestibular schwannoma concept	
1	exp Neuroma, Acoustic/ /
2	(vestibular schwannoma* or acoustic neuroma*).tw.
3	1 or 2
MRI concept	
4	exp magnetic resonance imaging/
5	(magnetic resonance imaging or MRI).tw.
6	4 or 5
Outcome concept	
7	(linear or rate or progression or growth or analysis or volume* or 3D* or size or segment* or diameter* or acquisition or sequence* or measure*).tw.
Combined concepts	
8	3 and 6 and 7
9	limit 8 to yr="2014 -Current"
Cochrane Central Register of Controlled Trials (CENTRAL)	38 articles
Vestibular schwannoma concept	
1	MeSH descriptor: [Neuroma, Acoustic] explode all trees
2	(vestibular schwannoma* or acoustic neuroma*):ti,ab,kw
3	#1 or #2
MRI concept	
4	MeSH descriptor: [magnetic resonance imaging] explode all trees
5	(magnetic resonance imaging or MRI):ti,ab,kw
6	#4 or #5
Outcome concept	
7	(linear or rate or progression or growth or analysis or volume* or 3D* or size or segment* or diameter* or acquisition or sequence* or measure*):ti,ab,kw
Combined concepts	
8	#3 and #6 and #7

**Table 2:** List of questions in Round 1 of the Delphi questionnaire

Ref	Question / Stem	Response Type	Items / Answer Options
Domain 1: Image Acquisition for VS Analysis and Management Planning			
1.1	What are the essential dedicated MRI acquisitions and other imaging sequences needed to plan VS management?	9-point Likert scale	• Thin section FSE T1w (2–3 mm axial sections) • Thin section FSE T1w pre and post gadolinium (2–3 mm axial sections) • Thin section FSE T2w (2–3 mm axial sections) • 3D high resolution T1w pre and post gadolinium (< 1 mm isotropic) • 3D high resolution T2w (< 0.8 mm isotropic) - gradient echo (CISS/c-FIESTA) or spin echo (SPACE/CUBE) • Dynamic contrast enhanced perfusion imaging (with gadolinium) • Dynamic susceptibility contrast perfusion imaging (without gadolinium) • PET • Other (free text)
1.2	Which single best sequence is optimal for VS measurement?	Single choice	• Thin section FSE T1w without gadolinium (2–3 mm) • Thin section FSE T1w pre and post gadolinium (2–3 mm) • Thin section FSE T1w post gadolinium (2–3 mm) • Thin section FSE T2w (2–3 mm) • 3D T1w without gadolinium (< 1 mm isotropic) • 3D T1w pre and post gadolinium (< 1 mm isotropic) • 3D T1w post gadolinium (< 1 mm isotropic) • 3D T2w (< 0.8 mm isotropic) - CISS/c-FIESTA or SPACE/CUBE • Other
1.3	In a scenario where AI tools may be validated on EITHER 3D high resolution T1w post gadolinium (typically <1 mm isotropic) OR high resolution T2w (typically <0.8 mm isotropic), which is the optimal sequence?	Single choice	• 3D high resolution T1w pre and post gadolinium (typically < 1 mm isotropic) • 3D high resolution T2w (typically < 0.8 mm isotropic) - CISS/c-FIESTA or SPACE/CUBE
Domain 2: Image Quality			
2.1	How strongly do you agree with the following statement? *"The AI algorithm should warn if the image quality appears sub-standard" (e.g. motion blur, slice thickness, tumour only partially visible)*	9-point Likert scale	
Domain 3: Measurement of Tumour			
3.1	Assuming the availability of validated automatic tools for tumour delineation, which MRI-derived size measurements should be routinely calculated for VS for longitudinal growth monitoring?	9-point Likert scale	• Two perpendicular linear axial extrameatal measurements • Two perpendicular linear axial extrameatal measurements and a craniocaudal measurement • Largest whole tumour linear axial measurement • Whole tumour volume • Extrameatal tumour volume • Separate extrameatal intrameatal volumes • Other (free text)
3.2	Which single (automated) measurement should be used to describe VS size?	Single choice	• Largest linear axial extrameatal measurement • Largest whole tumour linear axial measurement • Whole tumour volume • Extrameatal tumour volume • Other
3.3	Should the size of the tumour's anatomical intrameatal and extrameatal sub-regions be identified and measured?	Yes / No	
3.4	Should heterogeneous subregions within the tumour be identified/quantified? (e.g. cystic component, cystic necrosis, T2w heterogeneity)	Yes / No	
3.5	Which tumour sub-regions would be helpful to delineate?	9-point Likert scale	• Peritumoural cyst WITH a rim of enhancement • Peritumoural cyst WITHOUT a rim of enhancement • Intratumoural cyst • Non-enhancing T2 hyperintense area lateral to the tumour within the IAM (capping cyst) • Intratumoral cystic necrosis • Degree of T2w heterogeneity • Other (free text)
Domain 4: Interactive AI Tools			
4.1	How should clinicians be able to interact with AI software?	Multiple choice (select all that apply)	• Accept/reject AI generated tumour delineation • Correct segmentation (if tumour delineation unacceptable) • Correct linear measurement (if tumour delineation unacceptable) • Other
4.2	Who should be able to amend automated segmentations/measurements?	Multiple choice (select all that apply)	• Reporting radiologists - formally reject an AI segmentation • Reporting radiologists - adjust/change an AI segmentation • Specialist MDM radiologists - formally reject an AI segmentation • Specialist MDM radiologists - adjust/change an AI segmentation • Other MDT clinicians - formally reject an AI segmentation • Other MDT clinicians - adjust/change an AI segmentation • Other
4.3	How strongly do you agree with the following statement? *"If the interactive AI model requires fine-tuning, I would be comfortable with my interactions/feedback being used to help improve its performance"*	9-point Likert scale	
4.4	If standardisation is desired, should AI tools be able to "learn" from data at a local, national or international level?	9-point Likert scale	• AI tools should be able to "learn" using local data • AI tools should be able to "learn" using national data • AI tools should be able to "learn" using international data
4.5	How do you think an automated AI tool could impact workflow?	Free text (optional)	Open response
4.6	Are there any other important issues to consider regarding the implementation of an interactive AI tool?	Free text (optional)	Open response
Domain 5: AI Trustworthiness			
5.1	How strongly do you agree with the following statement? *"AI-derived measurements should be accompanied by a confidence measure"*	9-point Likert scale	
5.2	If a confidence measure is desired, which of the following methods should be employed?	9-point Likert scale	• Single confidence score for a given patient (e.g. percentage score trained on previous results/acceptance decisions) • Voxel-level confidence maps (e.g. 3D maps illustrating algorithm confidence in its segmentation) • Other (free text)
Domain 6: Predictive AI Tools			
6.1	Would it be helpful to have an automatically generated predictive score to reflect the likelihood of VS growth over a given time period?	Yes / No	
6.2	Would you accept a single predictive tumour score, or would you want to know the reasoning behind an AI-generated predictive score?	Single choice	I would accept a single score I would want to know the reasoning behind an AI-generated predictive score
6.3	How strongly do you agree with the following statement? *"An AI tool contraindicated for use in patients with multiple intracranial lesions (e.g. NF2-related schwannomatosis) would be problematic"*	9-point Likert scale	
Domain 7: MDT Communication			
7.1	What information would be helpful to provide to clinical members of the MDT?	9-point Likert scale	• Image thumbnail (e.g. axial MRI slice through tumour) • Single tumour measurement (e.g. linear, volume) • Graphical charting of tumour behaviour over time • Timing of intervention • Other (free text)
7.2	How should information be visualised and presented to clinical members of the MDT?	9-point Likert scale	• pdf summary • Digital platform (e.g. an app) • Other (free text)
Domain 8: Patient Communication			
8.1	What information would be helpful to provide to patients?	9-point Likert scale	• Image thumbnail (e.g. axial MRI slice through tumour) • Single tumour measurement (e.g. linear, volume) • Graphical charting of tumour behaviour over time • Timing of intervention • Other (free text)
8.2	How should patient information be visualised?	9-point Likert scale	• pdf summary • Digital platform (e.g. an app) • Other (free text)
Domain 9: Stereotactic Radiosurgery (SRS)			
9.1	How strongly do you agree with the following statement? *"Automated tumour delineation and volumetric tools should be incorporated into SRS planning software"*	9-point Likert scale	
9.2	Which of the following structures should be automatically segmented to aid SRS planning and delivery?	9-point Likert scale	• Vestibular schwannoma • Cochlea • Vestibule / semicircular canals • Facial nerve • Brainstem • Other (free text)
9.3	How strongly do you agree with the following statement? *"An AI tool should include automatically-generated dose plans"*	9-point Likert scale	
9.4	At what points in the SRS treatment planning pathway would it be best to integrate automated planning software?	9-point Likert scale	• Segmentation / contouring stage • Dose planning • Final treatment sign-off • Other (free text)

9-point Likert scale: 1 (Strongly disagree) - 9 (Strongly agree)

**Table 3:** Round 2 Delphi Survey Questions

Ref	Question / Stem	Response Type	Items / Answer Options
Domain 1: Image Acquisition for VS Analysis and Management Planning			
1.1	What are the essential dedicated MRI acquisitions needed to plan VS management?	9-point Likert scale	• 3D high resolution T1w pre and post gadolinium (< 1 mm isotropic) • 3D high resolution T1w post gadolinium (< 1 mm isotropic) • 3D high resolution T2w (< 0.8 mm isotropic) - gradient echo (CISS/c-FIESTA) or spin echo (SPACE/CUBE)
1.2	Which single best sequence is optimal for VS measurement?	Single choice	• 3D high resolution T1w pre and post gadolinium (typically < 1 mm isotropic) • 3D high resolution T1w post gadolinium (typically < 1 mm isotropic) • 3D high resolution T2w (typically < 0.8 mm isotropic) - CISS/c-FIESTA or SPACE/CUBE
1.3	Should AI tools be validated on BOTH 3D high resolution T1w post gadolinium (typically < 1 mm isotropic) AND high resolution T2w (typically < 0.8 mm isotropic)?	Yes / No	
Domain 2: Image Quality			
2.1	How strongly do you agree with the following statement? *"The AI algorithm should warn if the image quality appears sub-standard" (e.g. motion blur, slice thickness, tumour only partially visible)*	9-point Likert scale	
Domain 3: Measurement of Tumour			
3.1	Assuming the availability of validated automatic tools for tumour delineation, which MRI-derived size measurements should be routinely calculated for VS for longitudinal growth monitoring?	9-point Likert scale	• Two perpendicular linear axial extrameatal measurements and a craniocaudal measurement • Whole tumour volume
3.2	How strongly do you agree with the following statement? *"Whole tumour volume is the best single automated tumour measurement to describe VS size"*	9-point Likert scale	
3.3	How strongly do you agree with the following statement? *"Two perpendicular linear axial extrameatal measurements and a craniocaudal measurement should continue to be used in a transition period until serial volume measurements are established"*	9-point Likert scale	
3.4	Which of the following options is the single best volumetric metric of VS growth?	Single choice	• Volumetric increase > 20% • Volume Doubling Time (VDT) • Undetermined at present - further longitudinal studies using volumetric measurements required
3.5	How strongly do you agree with the following statement? *"Tumour anatomical sub-regions should also be identified and measured (e.g. intra-/extrameatal components)"*	9-point Likert scale	
3.6	How strongly do you agree with the following statement? *"Cystic change within solid tumour should be identified and quantified"*	9-point Likert scale	
Domain 4: Interactive AI Tools			
4.1	How strongly do you agree with the following statement? *"Any treating clinician (surgeons, radiologists, oncologists) should be able to accept/reject AI generated tumour delineation and correct segmentations/measurements"*	9-point Likert scale	
4.2	How strongly do you agree with the following statement? *"Only radiologists should be able to accept/reject AI generated tumour delineation and correct segmentations/measurements"*	9-point Likert scale	
4.3	Who should be able to amend automated segmentations/measurements?	Multiple choice (select all that apply)	• Reporting radiologists - formally reject an AI segmentation • Reporting radiologists - adjust/change an AI segmentation • Specialist MDT meeting radiologists (tumour board) - formally reject an AI segmentation • Specialist MDT meeting radiologists (tumour board) - adjust/change an AI segmentation
4.4	How strongly do you agree with the following statement? *"If the interactive AI model requires fine-tuning, I would be comfortable with my interactions/feedback being used to help improve its performance"*	9-point Likert scale	
4.5	How strongly do you agree with the following statement? *"AI tools should be able to learn from local and national data"*	9-point Likert scale	
Domain 5: AI Trustworthiness			
5.1	How strongly do you agree with the following statement? *"AI-derived measurements should be accompanied by a confidence measure"*	9-point Likert scale	
5.2	Which of the following methods should be employed to illustrate confidence?	9-point Likert scale	• Single confidence score for a given patient (e.g. percentage score trained on previous results/acceptance decisions) • Error bars for each feature assessed
Domain 6: Predictive AI Tools			
6.1	How strongly do you agree with the following statement? *"An automatically generated predictive score to reflect the likelihood of VS growth over a given time period would be clinically useful"*	9-point Likert scale	
6.2	How strongly do you agree with the following statement? *"I would want to know the reasoning behind an AI-generated predictive score"*	9-point Likert scale	
Domain 7: MDT Communication			
7.1	What information would be helpful to provide to clinical members of the multidisciplinary team (tumour board)?	9-point Likert scale	• Image thumbnail (e.g. axial MRI slice through tumour) • Single tumour measurement (e.g. linear, volume) • Graphical charting of tumour behaviour over time Timing of intervention
7.2	How should information be visualised and presented to clinical members of the MDT (tumour board)?	9-point Likert scale	• Automatically uploaded pdf summary attached to the patient's PACS entry • Automatically incorporated into the patient's radiology report on PACS • Automatically uploaded pdf summary attached to the patient's Electronic Health Records (EHR)
Domain 8: Patient Communication			
8.1	How strongly do you agree with the following statement? *"Patients should be provided with an automatically generated pdf summary graphically charting tumour behaviour over time"*	9-point Likert scale	
Domain 9: Stereotactic Radiosurgery (SRS)			
9.1	How strongly do you agree with the following statement? *"Automated tumour delineation and volumetric tools should be incorporated into SRS planning software"*	9-point Likert scale	
9.2	How strongly do you agree with the following statement? *"Automated SRS plan generation should be incorporated into SRS planning software"*	9-point Likert scale	
9.3	Which of the following structures should be automatically segmented to aid SRS planning and delivery?	9-point Likert scale	• Vestibular schwannoma • Cochlea • Vestibule / semicircular canals • Brainstem • Optic nerves and chiasm • Trigeminal nerve
9.4	How strongly do you agree with the following statement? *"An AI tool should include automatically-generated dose plans"*	9-point Likert scale	
9.5	At what points in the SRS treatment planning pathway should AI tools be utilised?	9-point Likert scale	• Segmentation / contouring stage • Plan generation • Plan evaluation

9-point Likert scale: 1 (Strongly disagree) - 9 (Strongly agree)

### Phase 2: achieving consensus through a Delphi process

To achieve a consensus for the standardized measurement of VS and use of AI in VS management, a modified Delphi method will be undertaken. This methodology consists of two sequential questionnaire rounds culminating in a final consensus meeting [17–20]. The Delphi survey was implemented online using Microsoft Forms (Microsoft Corp., Redmond, USA). An internal pilot was carried out amongst the steering committee, who are members of the BSBS, ESBS and ESHNR, to ensure clarity.

Stakeholders involved in in the management of VS will be recruited, including consultant skull base neurosurgeons, otolaryngologists, neuroradiologists and radiation oncologists, involved in the daily management of VS [21]. Stakeholders will be recruited through mass email via the intended professional groups – the BSBS, ESBS and ESHNR [22].

#### Delphi questionnaire round 1

Participants will evaluate each item using distinct scales:

1. Participants will determine the clinical relevance and importance of the item in the management of VS on a 9-point Likert-type scale. The scale is anchored as follows: 1–3 indicating ‘not at all important’, 4–6 indicating ‘important but not essential’ and 7–9 indicating ‘very important’.
2. Participants will also respond in a binary (Yes or No) or multiple choice format. Participants will be invited to share additional comments on each indicator as part of a free text response.

#### Calculating consensus about items

The distribution of ratings for each item relating to their importance will be graphed for visual analysis. Proportions of responses will be calculated across the three predefined categories: 1–3 (not at all important), 4–6 (important but not essential) and 7–9 (very important).

#### Items carried through to the second round of the Delphi

As per Carlson et al., consensus was defined a priori by the steering committee: moderate consensus as agreement of ≥ 67% and strong consensus as agreement of ≥ 80% [18]. Domains and questions will only be carried through to the second round of the Delphi where ≥ 67% of the sample rate it as top third of the importance scale (7–9 ‘very important’), with ≤ 15% rating it in the opposing third (1–3 ‘not at all important’). Qualitative analysis of the free-text suggestions will be performed independently by two reviewers.

#### Delphi questionnaire round 2

Participants who completed the initial questionnaire will be invited to the second round. This follow-up questionnaire will include items retained from the previous round, presented with the same rating scales.

Each participant will receive personalised feedback, displaying both their previous responses and those of the wider stakeholders. This feedback will include summary statistics such as measure of central tendency (e.g., mean, median or mode) and variability (e.g., standard deviation or interquartile range) for each item.

Participants will then be invited to review and, if desired, revise their ratings based on its importance.

### Phase 3: Delphi stakeholder consensus meeting

All participants who had taken part in previous rounds will be invited to a hybrid (in-person and online) consensus meeting on 22 January 2026 held in conjunction with the British Skull Base Society’s annual congress. Representatives from each key stakeholder group will attend the consensus meeting. During this meeting, data will be presented including frequency counts, measures of central tendency, and the distribution of scores. These results will again pertain to their (1) importance, and (2) whether it will be assessed, assuming the availability of validated assistive measurement tools.Following the presentation of the results, attendees will participate in focused discussions, deliberating each domain. Consensus will be reached through discussion. The revised document, listing the agreed guidance statements and recommendations for key areas requiring further research, will be circulated to participants for confirmation and minor wording suggestions. This process aims to ensure that the document accurately reflects decisions made during the consensus meeting. The final document will be distributed to all participants involved in the Delphi survey to seek their qualitative feedback.

### Public and patient involvement

The management of VS is made by a multidisciplinary team comprising skull base surgeons (neurosurgery and otolaryngology), neuroradiologists and radiation oncologists. Therefore patients and members of the public were not engaged in the development of this protocol.

### Ethics

Stakeholder recruitment will be conducted independently of National Health Service (NHS) organisations, primarily through relevant professional societies. The study was approved by the King's College London institutional review board (MRA-24/25-51552) [23]. Clinical trial number: not applicable.

## Discussion

AI-driven clinical decision support systems offer the potential to enhance patient outcomes by enabling both the standardisation and individualisation of VS management. The variability in methodologies used to guide VS management restricts the ability to synthesise evidence from the literature effectively, thereby undermining the validity of conclusions regarding treatment efficacy [24–26]. Whilst the development and deployment of AI tools for VS has the potential to standardise patient management, a consensus approach is required.

The 2003 International Consensus Meeting on Systems for Reporting Results in VS attempted to standardise research reporting to facilitate evidence syntheses and provide more valid information about treatment effects [4]. Due to the lack of available resources supporting volumetry at the time, the meeting had recommended using maximal linear measurements. Since then, studies have shown that volumetric measurement is a more sensitive and precise method of calculating the true size of the VS and is superior at detecting subtle growth [5, 6]. However, this time-consuming method, susceptible to inter-observer variability, has resulted in the lack of uptake of volumetric measurement into routine clinical practice [8]. From our systematic review, only 57.3% of studies measured volume by estimating from linear measurement or manual segmentations, and we had expected this figure to be greater in published studies compared to clinical practice.

Multiple research groups have developed fully automated AI frameworks capable of segmenting VS from MRI scan [8 ,27 ,28]. The first fully automated AI model demonstrated excellent accuracy achieving mean Dice scores > 93%, a score comparable to inter-observer agreement among clinicians [28]. AI-driven technology may offer the potential to expedite and standardise treatment decisions, potentially reducing the need for repeated imaging and clinical visits. Nonetheless, clinical adoption remains limited by concerns relating to the integration of AI into decision-making processes. Continued collaboration with both clinicians and patients is therefore required to ensure that the potential benefits of these technologies are effectively clinically translated.

Our Delphi protocol describes a multiphase approach for establishing a core set of imaging and measurement requirements for the management of VS, incorporating insights from a multidisciplinary panel of clinical experts actively involved in its management [29 ,30] We would like to highlight that the purpose of this consensus is only to identify which items are considered important and feasible for routine clinical use by multidisciplinary stakeholders. This process aims to standardise both management and subsequent outcome reporting of VS in research. The aim of the consensus was not to recommend volumetric thresholds for intervention. Indeed, further work is likely to be necessary to determine the clinical significance of volumetric change with respect to different tumour sizes. It should also be noted that while the resulting list of indicators will form a recommended minimum, it does not preclude the reporting of additional indicators relevant to the management of VS.

### Limitation

A limitation of the current work is that the Delphi questionnaire will be distributed only within the United Kingdom (UK) and Europe. However, a broader international perspective is incorporated through the identification of international studies published in English within the global literature. While the core measurement set is designed with practical relevance to NHS and European practice, it is acknowledged that stakeholders beyond this geographical region may prioritise different indicators.

### Impact and dissemination

By actively involving a multidisciplinary stakeholder steering group in the development of the Delphi study questions, and ensuring broad engagement from relevant clinical stakeholders throughout the questionnaire process, this study aims to enhance the future adoption of the resulting consensus recommendations and AI-driven clinical pipeline. Additionally, we will publish the Delphi study and guidelines in a peer-reviewed journal and present the results at relevant clinical and academic meetings.

## Conclusion

AI is transforming healthcare, and has the potential to positively impact the management of patients with VS. This study aims to reach a consensus to standardise the development and deployment of AI tools for VS management. A consistent approach in image acquisition, tumour measurement and the use of AI in VS management will improve patient care and support effective evaluation of emerging and novel treatments.

## Data Availability

No datasets were generated or analysed during the current study.
